# Intrusion of polyethylene glycol into solid-state nanopores†

**DOI:** 10.1039/c8ra00329g

**Published:** 2018-03-01

**Authors:** Yueting Sun, Chengliang Xu, Yibing Li

**Affiliations:** State Key Laboratory of Automotive Safety and Energy, Tsinghua University Beijing 100084 P. R. China syt@tsinghua.edu.cn

## Abstract

The intrusion of PEG aqueous solution into solid-state-nanopores upon mechanical pressure is experimentally investigated. By using hydrophobic nanoporous silica with a broad range of pore sizes, the characteristic size of PEG chains in water while penetrating nanopores is measured and analyzed, which increases with molecular weight and decreases with concentration of PEG. Its sensitivity to molecular weight is relatively limited due to nano-confinement. The inclusion of PEG as an intruding liquid imposes a rate effect on the intrusion pressure, and inhibits the extrusion from the nanopores.

## Abstract 2

The mechanism of PEG molecule penetration into nanopores upon mechanical pressure is understood.

## Graphical abstract

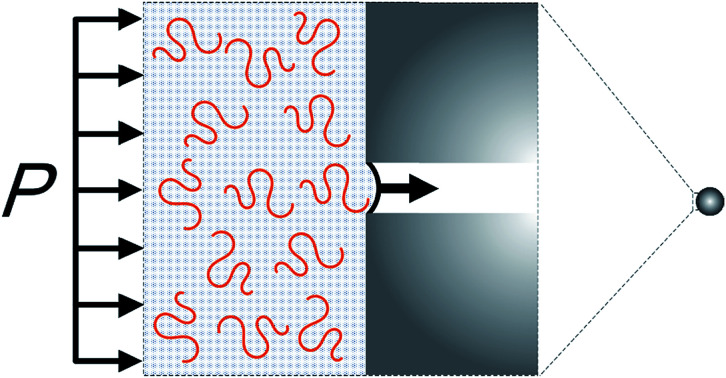

Poly(ethylene glycol) (PEG) is regarded as one of the world's most important water-soluble polymers owing to its numerous applications across various industries.^1^ Due to its biocompatibility and well-established safety profile (FDA approved), PEG is particularly popular in biomedical engineering.^2^ The conformations of PEG in solution (*i.e.*, helix or coil) have been extensively studied and well understood.^3^ Coil is the dominant conformation of PEG in water despite its short-range helicoidal sequences.^4^ While the knowledge for the bulk and macroscopic interfacial behavior of PEG has been well advanced, much less known is its behavior when penetrating the pores of nanometer.

Note that the intrusion and transport behavior of flexible polymer chains inside nanopores is of importance for many systems. Examples include molecule sequencing,^5,6^ drug delivery^7^ and molecular sieving,^8^*etc.* Nanopore confinement influences the conformation of polymer chains. If the pore diameter is comparable or smaller than the size of the polymer coil, there will be an entropic cost to hold the chain inside the pore because a number of possible configurations are lost.^9^ An external force may be applied to drive the entry and transport of polymer inside the pore, which can be electric field^10,11^ or concentration gradient.^12^

In this paper, we will present the first attempt to investigate the intrusion of PEG aqueous solution into nanopores upon mechanical pressure, to understand the behavior and configuration of PEG chains in water when entering nanopores. Solid-state nanopores are selected to enable reliable analysis on the characteristic size of PEG chains. Different molecular weights and concentrations of PEG solution are examined, due to the interest in understanding their influences.

The hydrophobic Fluka 100C_8_ reversed-phase nanoporous silica provided the solid-state nanopores investigated here, which had an average pore size of 7.8 nm with a standard deviation of 2.4 nm. The silica powder and PEG aqueous solutions were combined and sealed in a stainless-steel chamber (Fig. S1†). Driven by Instron 8872, a piston was compressed onto the specimen quasi-statically (0.5 mm min^−1^) so as to create a hydrostatic pressure for the intrusion of PEG solution into silica nanopores. Based on the recorded force and displacement data during the loading-unloading process, the *P*–Δ*V* curves (*i.e.*, pressure verses volume change of the specimen) were produced. Shown in Fig. 1 are the results at various PEG concentrations (*c* = 0–100 wt%) and molecular weights (*M*_w_ = 200–20 000 g mol^−1^).

**Fig. 1:**
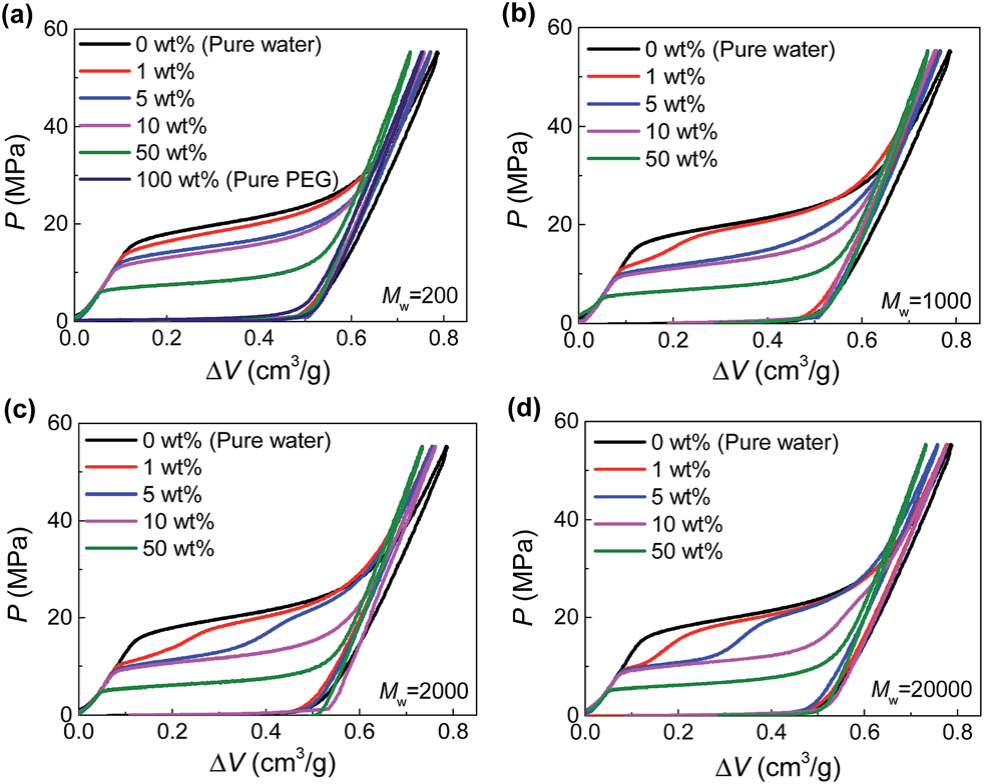
Pressurized intrusion of PEG solutions into silica nanopores at various PEG concentrations: (a) *M*_w_ = 200 g mol^−1^, (b) *M*_w_ = 1000 g mol^−1^, (c) *M*_w_ = 2000 g mol^−1^, (d) *M*_w_ = 20 000 g mol^−1^.

For the intrusion of pure water, due to hydrophobicity of the silica nanostructure, the system undergoes a linear elastic compression before the onset of water intrusion at a threshold pressure (∼16 MPa), coined intrusion pressure *P*_in_. The water intrusion corresponds to the plateau on *P*–Δ*V* curves which deviates from the elastic modulus of the system. Once all the nanopores are fully occupied by water molecules, another linear elastic part is obtained on the *P*–Δ*V* curve. For the unloading part, no obvious extrusion plateau can be observed, indicating the retention of the intruded water molecules.

Different from water, PEG is compatible with silica. Therefore they can spontaneously enter the nanopores without external pressure, as long as they geometrically fit the opening diameter. In that case, no intrusion plateau can be observed for the intrusion of pure PEG (as shown in Fig. 1a) since all the pore volume has been filled up before any pressure is applied. When PEG is mixed with water, owing to its wettability with silica and hydrophilicity as well, PEG serves as adhesive between silica and water. Therefore, *P*_in_ can be reduced since PEG can carry water molecules into nanopores with hydrogen bonds formed between their oxygen atoms and water molecules.^13^ As shown in Fig. 1a, *P*_in_ decreases from 16 MPa to 0 MPa when *c* increases from 0 to 100 wt%.

In the cases of larger PEGs, interesting two-step intrusion plateaus are observed (Fig. 1b–d) because only part of the plateaus drop down. Note that the silica investigated here has a broad pore size distribution, and the largest nanopores will be intruded first at relatively lower pressures. Therefore, the two steps of the plateaus are distinguished by the inclusion/exclusion of PEG for the penetrating liquid. That is, for larger nanopores that can accommodate PEG chains, *P*_in_ is lowered by the aforementioned intrusion promoting effect of PEG. However, when it comes to smaller nanopores which do not allow the entrance of PEG, *P*_in_ almost remains unchanged. In this way, the transition from the lower section to the higher section of the intrusion plateau actually reveals the characteristic diameter of PEG chains while penetrating the solid-state nanopores, noted as *d*_PEG_ in the following contents.

Then one important finding of *d*_PEG_ is its dependence on PEG concentration. Only relatively dilute PEG solutions exhibit two-step plateaus (generally *c* < 10 wt%, Fig. 1). Higher PEG concentrations will move the transition of the two steps to the right, indicating a decrease of *d*_PEG_. Further increase of the concentration will eventually give rise to a complete one-step low intrusion plateau, when PEGs become small enough to enter all the available nanopores, as shown in the cases of *c* = 50 wt%. According to a quantitative calculation of *d*_PEG_ based on the pore size distribution of silica and the position of the transition point between the two steps (see ESI†), *d*_PEG_ is found to decrease with *c* very significantly. Taking *M*_w_ = 20 000 g mol^−1^ as an example, *d*_PEG_ = 9.6 nm when *c* = 1 wt% while drops to 4.7 nm when *c* = 10 wt% (Table S1†).

Moreover, by comparing the results of different molecular weights at the same concentration (Fig. 2), we can find that *d*_PEG_ is also dependent on *M*_w_. The transition point between the two steps moves left with increasing *M*_w_, indicating that larger PEG molecules, as expected, have larger characteristic size *d*_PEG_. This also explains why in Fig. 1, *c* = 5 wt% is high enough for PEG of *M*_w_ = 1000 g mol^−1^ to enter all the nanopores (showing single-step plateau in Fig. 1b), while PEG of *M*_w_ = 20 000 g mol^−1^ at *c* = 10 wt% still cannot intrude the smallest nanopores (showing two steps in Fig. 1d). However, the sensitivity of *d*_PEG_ on *M*_w_ is rather limited. At the concentration *c* = 5 wt%, when *M*_w_ is increased for 10 times from 20 000 g mol^−1^ to 200 000 g mol^−1^, *d*_PEG_ just has a slight increase from 6.9 nm to 7.6 nm (Table S1†).

**Fig. 2:**
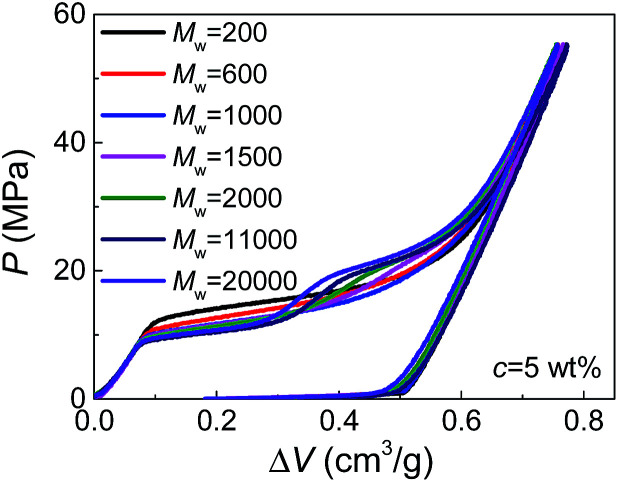
Pressurized intrusion of PEG solutions into silica nanopores with various molecular weights.

In bulk water without nano-confinement, the radius of gyration of PEG, *R*_g_, has been reported in open literatures, which also increases with molecular weight and decreases with concentration.^14,15^ This indicates the possibility of generalizing our existing knowledge on free PEG chains to nano-confinement conditions. However, it's worth noticing that the sensitivity of *d*_PEG_ on *M*_w_ is much lower than that of *R*_g_ in bulk water which is in the correlation of *R*_g_ ≈ 0.2*M*_w_^0.5^.^15,16^ This may be attributed to the geometrical re-organization of PEG chains upon nano-confinement during pressurized intrusion. Due to the flexibility of PEG chains, larger PEG molecules may still have similar sectional area to smaller PEG molecules, through their elongation along the axes of nano-channels. Besides, note that the values of *d*_PEG_ obtained here are quantitatively reasonable but slightly higher than the 2*R*_g_ or hydrodynamic diameters of PEG reported before, which should be caused by the repelling effect between nanopores and intruding liquids, and the consequent deviation of *d*_PEG_ from the nanopore diameter.^14,17^

Since PEG is a time-dependent polymer, the intrusion of PEG solution exhibits a rate sensitivity. When the intrusion rate is increased for 100 times (50 mm min^−1^), the intrusion performance of pure water has no significant change (Fig. 3). However, adding PEG introduces noticeable growth of *P*_in_ at the increased intrusion rate. Therefore, for the two-step intrusion plateau, the first section becomes higher, while the second section corresponding to pure-water-intrusion remains unchanged. A certain period of time, sometimes noted as persistence time, is required for PEG chains to transform their conformations, in this case, from the free state in bulk liquid to the nano-confined form.^17^ If the external pressure rises too fast, PEG chains tend to be temporarily frozen, resulting in an enhanced energy barrier for the conformation transition. Hence, the intrusion of the liquid containing PEG takes place at higher external stimulus, represented as increased *P*_in_ here. The rate sensitivity of characteristic size of PEG molecules is unclear yet.

**Fig. 3:**
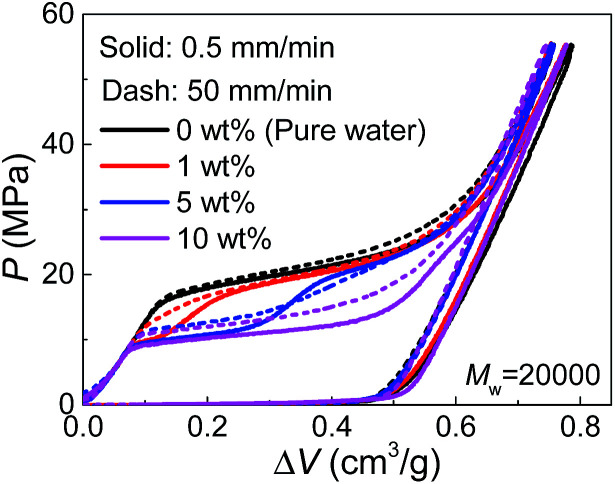
Pressurized intrusion of PEG solutions into silica nanopores at two different intrusion rates.

Due to their affinity to silica nanopores compared with water, PEG tends to inhibit liquid extrusion after external pressure is removed. According to our observations on the re-intrusion performance in the second loading cycles of the quasi-static intrusion (Fig. S2†), without PEG involved, a small fraction of water molecules (∼14%) will flow out of nanopores, but the addition of PEG can change it into a complete non-outflow performance. In order to have a better observation on the extrusion performance, we increased the environmental temperature during the tests to promote extrusion.^18^ As shown in Fig. 4, a complete extrusion is obtained for pure water when the temperature is elevated to 85 °C, so that its re-intrusion curve in the second cycle overlaps with the first cycle. This is related to the gas phase formation inside the nanopores which is more energetically favorable at elevated temperature.^19^ With the addition of PEG, the re-intrusion plateau in the second cycle becomes shorter, indicating that the extrusion of the previous cycle is inhibited. Intriguingly, the re-intrusion curve almost overlaps with the pure-water intrusion section of the first cycle. This reveals the fact that only the water molecules (that intrude smaller nanopores) flow out of nanopores upon unloading. For the larger nanopores that accept PEG chains, all the liquid molecules are locked inside.

**Fig. 4:**
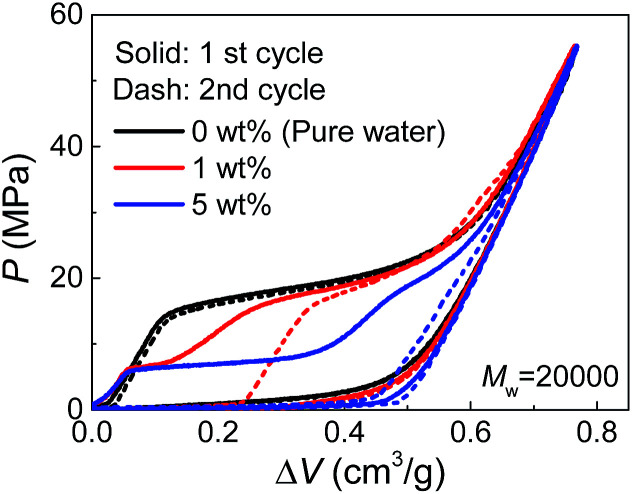
Quasi-static cyclic intrusion of PEG solutions into silica nanopores at 85 °C.

The above observations facilitate the understanding on the intrusion behavior of PEG solution into solid-state-nanopores. By using nanopores having a broad range of pore size, the characteristic size of PEG chains is measured, which proves to decrease with PEG concentration and increases with molecular weight. Importantly, the sensitivity of characteristic size on molecular weight is quite limited probably due to the nanoscale constraint. The intrusion of PEG solution proves to be rate dependent and without extrusion. Besides, it's also worth mentioning that the pressure-induced liquid intrusion observed here actually dissipates substantial mechanical energy (∼10 J g^−1^), and its two-step performance (which can be controlled by PEG concentration and molecular weight) promises a smart ‘step-by-step’ energy absorption system that can cope with different impact levels. Upon moderate impacts, the first low-pressure section can absorbs energy and lowers the impact force; upon severe impacts, the subsequent high-pressure intrusion can be activated to absorb more intensive mechanical energy and keep the transmitting wave at low amplitude.

## Conflicts of interest

There are no conflicts to declare.

## Supplementary Material

RA-008-C8RA00329G-s001
