# Supplementary material for: Intrusion of polyethylene glycol into solid-state nanopores
Source: RSC Adv. 2018 Mar 1;8(17):9070–3. doi: 10.1039/c8ra00329g (PMC9078677; doi:10.1039/c8ra00329g)
Supplement: RA-008-C8RA00329G-s001 [file RA-008-C8RA00329G-s001.pdf]

Electronic Supplementary Material (ESI) for RSC Advances.
This journal is © The Royal Society of Chemistry 2018

Supplementary Information

Intrusion of polyethylene glycol into solid-state nanopores

Yueting Sun,* Chengliang Xu, Yibing Li

State Key Laboratory of Automotive Safety and Energy, Tsinghua University, Beijing, 100084, P.R. China

* Corresponding author. Email: syt@tsinghua.edu.cn (Y. Sun)

Experimental section

Sample preparation

The Fluka100 C_8 reversed-phase nanoporous silica gel was obtained from Sigma-Aldrich (Product No. 60755) and used as received in white powder form. It had an average pore size of 7.8 nm and a standard deviation of 2.4 nm. Its specific pore volume and pore surface area were 0.55 cm^3/g and 287 m^2/g respectively. Its particle size ranged from 10 to 35 μm. PEG of different chain lengths (with 200-20000 in molecular weight) were obtained from Sinopharm Chemical Reagent Co.,Ltd, and used as received, either in liquid or solid form. PEG was combined with water to form a uniform PEG aquaous solution by mechanical stirring. Various concentrations (0-100 wt%) of PEG solution were obtained by using different weight ratio of PEG to water.

200 mg silica powder was placed into the chamber shown in Figure S1. Then around 2 ml PEG aqueous solution was also put inside and then sealed by a piston with precisely fitted sealing rings. Both the chamber and piston were made of stainless steel 304, and the diameter of the piston was 12.7 mm. The initial length of the specimen inside the chamber (i.e., the mixture of silica and PEG solution) was 4.0 mm.

Pressure-induced liquid intrusion tests

The intrusion tests were conducted on Instron 8872. The piston was compressed into the chamber to creat hydrostatic pressure onto the specimen inside. For quasi-static compression, 0.5 mm/min was selected as the loading rate, while for higher loading rate tests (Figure 3a), the moving velocity of the Instron crosshead was increased to 50 mm/min. 56 MPa (7kN) was selected as the peak pressure, at which all the available nanopore volume was expected to be occupied by the liquid. Then the piston started to move back at the same velocity for unloading. For the tests at increased temperatures (Figure 3b), a temperature controlled water bath (AS ONE EO-200RD) was used to create and keep a stable enviornmental temperature for the specimen during the loading-unloading process.

During the tests, the reaction force on the piston F and the displacement of the piston d were recorded by the Instron machine. Then the pressure of the specimen equaled $P = F/A$, and its specific volume change was $\Delta V = (A \cdot d)/m$, where A was the sectional area of the piston (126.6 mm^2) and m was the mass of silica (200 mg). Accordingly, P-ΔV curves were plotted for the analysis of PEG intrusion. The length of the intrusion plateau indicates the total pore volume available for liquid intrusion, which was measured around 0.5 cm^3/g, very close to its theretical value 0.55 cm^3/g.

Calculation methods

Calculation of effective size of PEG molecules

The effective size of PEG molecules d_{PEG} is calculated based on the pore volume fraction associated with the two steps of the intrusion plateau. Assume that the lower section takes $l/(l+h)$ of the total plateau and higher section takes the rest part $h/(l+h)$, then d_{PEG} should be larger than $h/(l+h)$ of all the nanopores. Since the pore size follows the normal distribution function $N(\mu, \sigma^2)$ with the average diameter μ=7.8 nm and the standard deviation σ=2.4 nm, then we can obtain d_{PEG} by the corresponding cumulative distribution function $P(X \leq d_{PEG}) = h/(l+h)$. For simplicity, the transition point between the two steps of the intrusion plateau is taken as the middle point of the transition zone between the two steps of intrusion plateaus. Calculation results of PEGs under various conditions are listed in Table S1. Note that only the P-ΔV curves with two clear steps of plateaus are used for calculation.

Calculation of energy dissipation

The mechanical energy dissipation is calculated according the area enclosed by the loading and unloading curve on the P-ΔV plots. The intrusion of pure water dissipates an energy of 11.27 J/g. Due to the partial drop of the intrusion plateaus, the addition of PEG will decrease the energy dissipation. For instance, the solution of PEG20000 at a concentration of 5 wt% has an energy dissipation density of 8.35 J/g.

Table S1. Calculated characteristic diameter of PEG molecules.

M_w (g/mol)	***c*** **(wt%)**	$h/(l+h)$	d_{PEG} (nm)
20000	1	0.778	9.6
20000	5	0.474	7.6
20000	10	0.098	4.7
11000	1	0.778	9.6
11000	5	0.41	7.3
2000	5	0.35	6.9

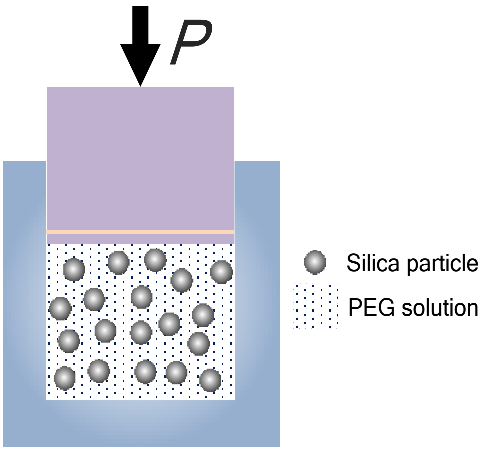

Figure S1. Experimental setup

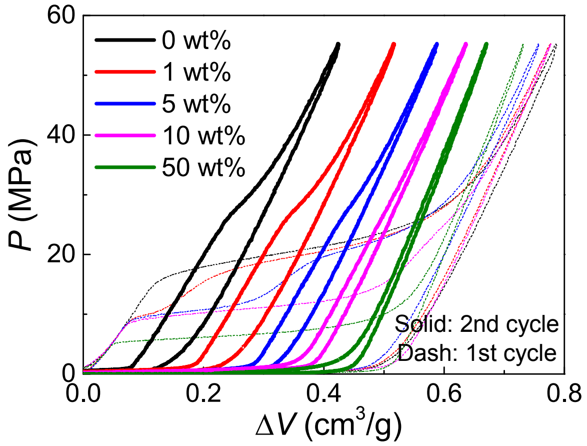

Figure S2. Two cycles of the pressurized intrusion of PEG solutions into silica nanopores at various concentrations.
